# Understanding Clinicians’ Informational Needs for AI-Driven Clinical Decision Support Systems: Qualitative Interview Study

**DOI:** 10.2196/85228

**Published:** 2026-03-12

**Authors:** Simone Mingels, Hannah Piehl, Madeline Therrien, Ekaterina Akhmad, Anniek van Hienen, Johan van Soest, Laura Hochstenbach, Andre Dekker, Olga Damman, Rianne Fijten

**Affiliations:** 1 Department of Radiation Oncology (Maastro), GROW Research Institute for Oncology and Reproduction, Maastricht University Medical Centre+, Maastricht University, Paul Henri Spaaklaan 1, Maastricht, 6229 EN, The Netherlands, +31 (0)43 38 81863; 2 Brightlands Institute for Smart Society (BISS), Faculty of Science and Engineering, Maastricht University, Heerlen, The Netherlands; 3 Department of Health Services Research, Care and Public Health Research Institute (CAPHRI), Maastricht University, Maastricht, The Netherlands; 4 Department of Public and Occupational Health and Amsterdam Public Health Research Institute, Quality of Care, Amsterdam UMC location VUmc, Amsterdam, The Netherlands

**Keywords:** artificial intelligence, AI implementation, delivery of health care, informational needs, reporting standard, transparency, co-creation

## Abstract

**Background:**

Advancements in artificial intelligence (AI) are transforming health care, particularly through AI-driven clinical decision support systems (AI-CDSS) that aid in predicting disease progression and personalizing treatment. Despite their potential, adoption remains limited due to clinician concerns about algorithm misuse, misinterpretation, and lack of transparency.

**Objective:**

This qualitative study explores the informational needs and preferences of clinicians to better understand and appropriately use AI-CDSS in decision-making. In parallel, this study explores AI experts’ perspectives on what information should be communicated to enable safe and appropriate use of AI-CDSS.

**Methods:**

A qualitative description design study was conducted using semistructured interviews with 16 participants (8 clinicians and 8 AI experts). Discussions focused on experiences with AI, informational needs, and feedback on existing reporting standards, including Model Cards, Model Facts, and the Transparent Reporting of a multivariable prediction model for Individual Prognosis Or Diagnosis–Artificial Intelligence (TRIPOD-AI) checklist. The transcripts were analyzed through codebook thematic analysis.

**Results:**

Four key themes were identified: (1) clinicians need clear information on training data, its origin, size, and inclusion and exclusion criteria, to judge model applicability; (2) performance metrics must go beyond the area under the curve (AUC) and be clinically relevant to support informed decisions; (3) limitations and warnings about inappropriate use should be specific and clearly communicated to prevent misuse; and (4) information should be presented in layered, customizable formats within existing clinical software, avoiding unnecessary jargon, and allowing optional deeper explanations. While each of the reviewed reporting standards offered strengths, none were considered sufficient alone. Participants recommended a combined and clinician-centered approach to information delivery. Alignment of reporting standards with clinical workflows and decision thresholds was thought to be crucial to bridge the usability gap.

**Conclusions:**

To improve AI-CDSS adoption in clinical practice, reporting standards must be designed for better clinician comprehension and usability. Enhancing transparency, particularly regarding training data and performance, can likely help clinicians assess AI-CDSS more effectively. Information should be delivered in an accessible, layered format, fitting clinical workflows. Co-creation with clinicians throughout AI-CDSS development was a cross-cutting theme, highlighting its importance in ensuring tools are not only technically sound but also practically usable. Future research should explore how to structurally report on performance and validation metrics for clinician understanding and assess the impact of information provision on AI-CDSS adoption.

## Introduction

Advancements in artificial intelligence (AI) are rapidly transforming the health care sector [1]. AI has the potential to aid clinicians and patients in comparing various treatment options by predicting future events based on individual patient characteristics to determine which treatment would benefit the patient the most [2]. Implementing these AI-driven clinical decision support systems (AI-CDSS) offers the potential for highly personalized medicine by predicting disease survival probabilities and potential treatment side effects [3,4]. Despite its potential, achieving sustained adoption of AI-CDSS in routine clinical care frequently encounters challenges [5].

One of these challenges is clinicians’ resistance to adopting AI-CDSS, which is exacerbated by their concerns about the high potential risks to patient safety and quality of care [6,7]. Among clinicians, there can be a lack of in-depth knowledge of how algorithms are constructed and how they generate predictions and recommendations [8]. This can make clinicians unprepared to assess if the algorithm is usable within their specific clinical setting, which can cause (unintended) misuse of algorithms [9-11]. An example of such misuse can be found in the study by Zhao et al [12], where the INFLUENCE tool (IKNL) was applied to guide decisions about primary therapy. However, this tool was originally designed to estimate individual, time-dependent risks of recurrence or metastasis in patients with breast cancer who have already completed curative treatment [13]. Using the model outside its intended context led to inadequate treatment recommendations [14].

The lack of knowledge that can exist amongst clinicians is not surprising, since there is an overall inadequate adherence to reporting standards within the AI development field [15-17]. Even when developers adhere to these reporting standards, the reports are often too technical for clinical end users to adequately understand the potential risks or the intended use of the models [18,19]. Descriptions of AI-CDSS, such as intended use and target population, can help clinicians better understand the meaning of the outcome produced by a model before acting on its recommendation, which can prevent mistakes made due to misinterpretation of the outcome [20].

To stimulate reporting of prediction models in published literature, several initiatives have been introduced [21]. The Transparent Reporting of a multivariable prediction model for Individual Prognosis Or Diagnosis–Artificial Intelligence (TRIPOD-AI) statement is a set of recommendations for the reporting of studies developing, validating, or updating a prediction model and is commonly used and/or required by journal publications [22-24]. In addition, other guidelines such as the CONSORT-AI (Consolidated Standards of Reporting Trials extension for Artificial Intelligence) [25], Developmental and Exploratory Clinical Investigations of Decision Support Systems Driven by Artificial Intelligence (DECIDE-AI) [26], and SPIRIT-AI (Standard Protocol Items: Recommendations for Interventional Trials involving Artificial Intelligence) [27] were created. However, the focus of these reporting standards is on transparent reporting within clinical trial reports or protocols, not on improving understanding of AI for clinicians [28,29]. Furthermore, these reporting standards are developed to report on an AI model and not the environment or software system in which it is operating [30].

In an attempt to further standardize reporting of AI model information, Google established the concept of “Model Cards”, which is comparable to providing a recipe and nutritional facts to a meal [31]. Through Model Cards, developers provide a one- or 2-page record highlighting characteristics of training data, intended use cases, and performance [32]. This Model Card can boost conformation to reporting standards but does not contain actionable information and guidance for applying AI in clinical practice. Sendak et al [33] reconstructed the idea of Model Cards using the concept of drug fact boxes. Drug fact boxes are used to understandably communicate benefits and risks associated with medications to patients [34]. These concepts combined resulted in “Model Facts” [33], an overview to “collate relevant, actionable information in a compact overview to ensure that clinicians know how, when, how not, and when not to use model output in their clinical decisions” [33].

Although the Model Facts concept is not thoroughly tested yet, the initiative has sparked a discussion on how to effectively communicate the potential risks of AI models to health care professionals [33]. This highlights a broader need to understand what information clinicians require in order to evaluate and appropriately use AI-driven recommendations in their clinical decision-making. Reflecting these developments, the study investigates the following questions:

- What are the informational needs and preferences of clinicians for understanding and appropriately using AI-driven recommendations on medical decision-making?
- What information, according to AI experts, should be communicated to clinicians to enable their safe and appropriate use of AI-CDSS?

## Methods

### Study Design

This qualitative description design study [35] was conducted using semistructured interviews to explore how to effectively communicate information about AI-CDSS in medical decision-making. Interviews were conducted among two groups: (1) clinicians, to understand their preferences and informational needs, and (2) AI experts, to gain insights into what experts consider necessary for clinicians to safely and appropriately use AI tools. For this study, AI expertise was established based on practical professional experience rather than a specific formal education in AI. The expert group consisted of individuals with direct experience in developing or implementing AI models for or in the health care sector. The inclusion of both groups was based on the assumption that informational needs go beyond the clinicians’ expressed preferences, but also expert-identified information is necessary for informed and safe use of AI in clinical settings. This article was written in accordance with the COREQ (Consolidated Criteria for Reporting Qualitative Research) checklist (Checklist 1) [36].

### Recruitment of Participants

Study participants were recruited by convenience and snowball sampling between July and November 2024. A target sample size of 8 participants per group was predetermined. This fixed quota was established to accommodate the limited availability of clinicians while ensuring balanced representation between the 2 groups. Consequently, recruitment was concluded upon reaching this target rather than based on theoretical saturation. Potential participants were identified through professional networks associated with the research team and targeted searches on LinkedIn (Microsoft). The potential participants were contacted by email, accompanied by study information detailing the study’s objectives, procedures, and confidentiality measures for the recording of the interviews. The interviewees interested in participating filled in a consent form and sent back an email to schedule a meeting, either online or face-to-face. Inclusion criteria required participants to be either (1) practicing clinicians in the Netherlands or (2) AI experts, entailing those with experience in developing and/or implementing diagnostic or treatment recommendation AI models within the health care setting. Participants who had less than 2 years of work experience were excluded.

### Data Collection

The semistructured interviews were conducted by a researcher (SM), who is proficient in both Dutch and English. The interviews followed an interview guide (MultimediaAppendices 1  2), which contained different questions for clinicians and AI experts, each consisting of 4 parts (Table 1). The guides were translated to English for those with no sufficient command of the Dutch language by researchers SM and EA.

**Table 1.** Outline of the interview guide topics for clinicians and artificial intelligence (AI) experts regarding AI-driven clinical decision support systems (AI-CDSS) use.

Clinicians	AI^a^ experts
Demographic information	
Experience with AI in care settings	Experience developing AI for care settings
Current received information about AI models	Provided information concerning AI models
Information they would prefer to receive	Information they think they should provide
Examples of the Model Card, TRIPOD-AI,^b^ and Model Facts were shown, and participants were requested to provide feedback concerning amount, structure, and comprehensiveness of the information^c^	

a AI: artificial intelligence.

b TRIPOD: Transparent Reporting of a multivariable prediction model for Individual Prognosis Or Diagnosis–Artificial Intelligence.

c This part was equal for clinicians and AI experts. Demographic information was also available for both the clinicians as well as for the AI experts.

In part one, the participants were asked demographic questions concerning their age, medical or technical specialization, and work experience history. In the second part, clinicians were asked about their experiences with AI in the health care setting and about instructions they received before using AI. AI experts received questions about their experience in developing AI for clinical use and which information they provided to the end users. Part three contained questions about which information they would ideally either receive as clinical end users or provide as AI developers.

In the last part, all participants received an example of a Model Card [32], Model Facts [33], and the TRIPOD-AI checklist [22,24] (Multimedia Appendix 3). The formats were discussed sequentially: participants reviewed one example and provided immediate feedback on its information before moving to the next. This stepwise, fixed sequence ensured that each format was evaluated individually. These three formats were selected because they originate from distinct contexts and serve different purposes. Model Cards are intended for communication among developers and are therefore more technical in nature. In contrast, Model Facts are specifically designed for clinicians. The TRIPOD-AI checklist is a reporting standard on AI models in academic publications. To create these examples, a Bayesian network was selected from a public repository, as it provided a balanced level of complexity, being more complex than a linear regression but still more transparent than a deep learning model. One researcher (EA) completed each format based on the available model information. To preview a variation in communication styles, the same type of information was presented in bullet points in one format, while in another format, it was written in narrative form, and yet another displayed this information in a table. After examining the templates, participants were asked to provide feedback on the amount, structure, and comprehensiveness of the information listed in these examples.

### Research Group

SMcandidate in Clinical Data Science (CDS) at Maastricht University, working in a department that is generally tech-positive, which may have shaped her perspectives on AI in health care. Over the course of this research project, SM engaged with different paradigms, initially approaching research from a postpositivist stance but increasingly shifting toward a constructivist orientation. This perspective informs the study’s recognition that a single “ground truth” of information provision is not attainable and that knowledge is context-dependent and co-constructed.

HP is a PhD student researching trust in AI within CDS. MT is a PhD student at Maastro Clinic, researching risk communication and shared decision-making in breast cancer radiation therapy, with a focus on patient decision aids. EA is a PhD student within CDS investigating AI model descriptions based on the model representation standards and requirements. AvH is a PhD student in shared decision-making within radiation oncology (CDS and Maastro), with advanced training in qualitative methodology, and advised SM on qualitative methods. RF is an assistant professor in AI and clinical decision-making within CDS and Maastro and supervises the PhD candidates.

### Data Analysis

After data collection, recordings were transcribed verbatim and analyzed according to codebook thematic analysis [37] using the Atlas.ti software (version 25.0.1; ATLAS.ti Scientific Software Development GmbH). This method helped identify common themes and topics that were mentioned repeatedly in interviews. The analysis process started with open, inductive coding by a researcher (SM). As no predefined coding framework was used, codes were created to descriptively summarize the underlying meaning of the selected text segments. This initial phase resulted in the construction of a preliminary codebook. Two other researchers (MT and HP) independently applied codes from the codebook constructed by the first researcher (SM) to a subset of the interviews (n=4). Disagreements in codes were resolved by revisiting the transcripts to verify context and debating code definitions until consensus was reached. This iterative process changed the codebook by renaming codes, refining code definitions, ensuring consistent application of the codes, and merging overlapping codes. These codes were later grouped into general themes by identifying clusters within assigned codes for clinicians (Multimedia Appendix 4) and for AI experts (Multimedia Appendix 5). A member check of the analysis was not conducted to avoid influencing participants’ perspectives in potential follow-up research.

### Ethical Considerations

Before the start of the study, the Research Ethics form of the Faculty of Health, Medicine, and Life Sciences (FHML-REC, FHML/HDT/2024.022.) was evaluated, and ethical approval was granted. Prior to the interviews, all participants received an information letter detailing the study’s objectives and data handling procedures. Written informed consent was obtained from all participants for both participation and audio recording. To ensure confidentiality, all transcripts were pseudonymized, and any identifying information was removed. Participants were provided access to both their audio recording and the corresponding transcript, which they could review at any time. Participants received no compensation for their participation.

## Results

### Participant Characteristics

In total, 26 clinicians and 11 AI experts were invited to participate. Among invited clinicians, 8 declined due to lack of time, while 10 did not provide a reason. Three invited AI experts also did not provide a reason for nonparticipation. Between August and November 2024, 16 interviews were conducted with clinicians (n=8) and AI experts (n=8). Half of the clinician interviews were held through Microsoft Teams, and the other half were face-to-face, whereas most AI expert interviews were face-to-face (n=6), 2 AI expert interviews were held in English, while all remaining interviews, including all clinician interviews, were conducted in Dutch. Interviews lasted between 30 minutes and an hour. All participating clinicians practiced within tertiary care settings, employed either at academic medical centers or a specialized institute. Concerning prior experience with AI among clinicians, all reported having encountered AI tools in their professional practice. The majority (n=5) had experience with prediction models; some (n=2) had worked exclusively with AI-driven imaging applications or generative AI in the form of chatbots (n=1). Participant characteristics are presented in Table 2.

Through conducting thematic analysis, 4 key themes were identified that clinicians and AI experts expressed as essential for effective communication and use of AI-CDSS. These themes include (1) understanding the target population, which refers to the need to understand the data on which the model was trained; (2) clinically meaningful outcomes, focusing on how model reliability and clinical relevance are communicated; (3) warnings, limits, and safe use, highlighting the importance of clearly defining boundaries, limitations, and misuse risks; and (4) accessible design, which addresses the structure and presentation of content. These themes and subthemes can be found in Table 3 and are discussed in detail below.

**Table 2.** Demographic and professional characteristics of the 16 participants interviewed between August and November 2024 in the Netherlands.

Characteristics	Clinicians (n=8)	AI^a^ experts (n=8)
Gender		
Men	6	5
Women	2	3
Age (years), median (range)	44 (29–59)	37 (28–48)
Work experience (years), median (range)	14 (2–22)	11 (3–21)
Specialties or roles (n)	Internal medicine (2) Oncology (2) Gastroenterology (2) Cardiology (1) Radiology (1)	Medical imaging AI (3) Innovation and implementation management (3) Data infrastructure and FAIR^b^ principles (2)

a AI: artificial intelligence.

b FAIR: Findable, Accessible, Interoperable, and Reusable.

**Table 3.** Overview of themes and subthemes derived from interviews with clinicians and artificial intelligence (AI) experts regarding informational needs and preferences for using AI-driven clinical decision support systems (AI-CDSS).

Theme	Subthemes
Understanding the target population	Origin of the data Class imbalance Inclusion and exclusion Training data size
Clinically meaningful outcomes	Difficult AUC^a^ Uncertainty bands Performance graphs and comparative visualizations
Warnings, limits, and safe use	Underused, poorly highlighted, and filled in too vaguely Active warnings
Accessible design	Available at all times Language barrier Customizable or layered information provision

a AUC: area under the curve.

### Understanding the Target Population

A central theme expressed by most clinicians and experts was the ability to compare an individual patient during consultation with the training population for the AI model. This was fundamental for clinicians to evaluate whether the recommendations from the model would be reliable in a specific clinical context or not.

*What I think they should know is on which population it (AI-CDSS) is based. So for which patient are they allowed to use it and for which patient are they not. If they have a new patient in front of them, when does it (the patient) belong within the population of the model.*

[AI Expert 2]

Initially, participants described this need as knowledge about the origin of the data, more specifically why, where, and how the data were collected. Clinicians stressed that this way, they could extrapolate whether this reflected local populations, which was especially important when the model was not locally validated.

*Where is the population from? This is clearly a Dutch population, I can extrapolate this to our own thing, but I would want to see the patient characteristics.*

[Clinician 4]

Although the concern about the representativeness of the data and its relevance to local populations was present from the start, it became more concrete when participants reviewed the TRIPOD-AI example. Clinicians remarked that the section “class imbalance” was of great importance to make the comparison. However, the way it was written in the example felt overwhelming and too technical. Solutions for this were given, such as a table or graph to show the class imbalance, but also a digital and interactive solution for training data comparisons. AI experts already stressed the importance of providing information about the class imbalance before being shown the TRIPOD-AI example.

(*Showed class balance in TRIPOD-AI example) Yes, basically yes, yes. Look, and if you see that they’re all N1 patients, then is it also still suitable for N2? If your patient happens to be N2, so all those kind of things. Yeah I think that’s really important to take into account.*

[Clinician 5]

Later in the TRIPOD-AI document, the inclusion and exclusion graph for the training data, which is often provided in papers, was shown. Clinicians stressed that without understanding who was systematically left out of the training data, whether by design or by oversight, they felt the context to interpret the applicability lacked.

*For example, it doesn’t apply to pregnancy or people who are overweight with a BMI over 30, I’m just saying something. Because otherwise, I think you lose sight of that when you’re working in practice.*

[Clinician 7]

Finally, both clinicians and AI experts noted that information about the training data size was missing in the Model Facts example. This was, however, crucial information according to clinicians, since a great size of the training data is associated with better model performance.

*And actually, I’d want to know how many patients there were, like I’d want to know for reliability, how many patients were in the model so I can estimate how good it is.*

[Clinician 4]

### Clinically Meaningful Outcomes

Another key theme was the need for clear evidence of the performance and reliability of the model. Both groups stressed that for an AI-CDSS to be integrated into clinical practice, its performance must be demonstrably robust and easily interpretable. However, the manner in which the performance should be presented, as well as when it was acceptable, was not agreed upon by participants.

Clinicians, in particular, emphasized the importance of understanding what the reported performance metrics mean in practice. They noted that values like area under the curve (AUC), while technically informative, do not always translate into clinical insight without additional context. Some participants mentioned a desire for performance measures to align more closely with clinical outcomes, like sensitivity and specificity and their relevant thresholds. Clinicians and AI experts suggested that these measures can also easily be translated into statistical language such as false positives or negatives, which was thought to be more familiar to clinicians.

*The AUC just gives an overall picture of whether it is a good predictive model. But if it’s 0.88 and the balance between sensitivity and specificity for your specific question isn’t optimal, then it still isn’t a good model.*

[Clinician 3]

Experts mentioned that besides the performance measures, there should also be a focus on providing an uncertainty band to clinicians. This should give insights into when the model is handling an edge case. AI experts noted that in the AI development at the time of this writing, developers chase a high-performance score such as the AUC but might overlook clinical utility or model robustness by doing so.

*We often try to get that AUC as high as possible, but that applies to the entire population, so maybe we should start saying, maybe we need to develop models that actually filter out the worst-off patients or the ones who do the best.*

[AI expert 2]

Ultimately, while clinicians overall claimed to be familiar with performance metrics, they expressed that information on model validation often remains buried in dense technical language or tables. However, to be actionable in a clinical setting, they emphasized that information must be presented in a more structured and visually digestible manner, such as performance graphs and comparative visualizations. Besides presenting the performance metrics, clinicians expressed a desire to know when they could use an outcome to make a treatment decision, in other words, when it was “good enough” to rely on.

*Yes, these are always somewhat abstract concepts if you don’t work with them daily. And yes, each time I can reason it out – like, what it means – but I can’t immediately say whether it’s good or not, right?*

[Clinician 5]

### Warnings, Limits, and Safe Use

An important theme for both clinicians and experts was the need for clear warnings and boundaries for AI-CDSS use. Explicit indicators or limitations were desired, especially when the system may be applied in situations that fall outside its intended use or scope.

Participants praised that limitations and inappropriate uses were included in the presented reporting standards. However, many AI experts emphasized that these sections were often underused, poorly highlighted, and filled in too vaguely. AI experts also mentioned that biases and limitations of the model are difficult to formulate and fill in with only their background as developers. They mentioned that defining these biases and formulating necessary warnings for misuse of the model should be done in interdisciplinary settings. They also emphasized that misuses of the model can be identified during prototype testing or practice sessions.

*So I would say that, yes, this requires critical evaluation. The problem, I think, is that if someone developing the model doesn’t have much clinical background, it becomes very difficult for them to judge what is critical and what should be reported.*

[AI expert 3]

Besides explicitly mentioning these limitations, participants also stressed that more active warnings should be included. AI experts mentioned that it would be safest to lock down the scope of the AI-CDSS within the software, such as limiting input variables or blocking predictions outside the scope. However, clinicians had varying opinions about this, with some arguing that they can decide on the model scope themselves. Another suggestion made by AI experts to warn clinicians about the correct use of an AI-CDSS was to provide pop-ups when using the model out of scope. However, opinions amongst clinicians were divided, with concerns regarding pop-up tiredness and the pop-up not being read or respected. Similarly, they thought that disclaimers were likely not to be read or forgotten about during the clinical use of the model.

*There are people in their 60s who function at the level of someone over 70, or even over 80, so to speak. And there are people who are 72 where you think, well, you actually function better than your calendar age would suggest. So it’s all a matter of interpretation. And I think that this is something that should be left to a clinician.*

[Clinician 8]

### Accessible Design

Participants emphasized that how information is presented is just as critical as the content itself. Even when essential data about the system is available, it may be ignored or misunderstood in clinical settings if it is not accessible, clearly structured, or presented in a usable format.

First, the information needs to be available at all times while using the AI-CDSS, according to participants. They stressed that information should preferably be integrated within the software. However, it was also thought to be important to distribute information in different ways, such as presentations, workshops, and emails. According to participants, clinicians should also familiarize themselves with the model, which can be done through e-learning and practice sessions, which is important for retention of their understanding of the model.

*But if you, for example, give a presentation and a doctor can actually use your model that same day, then that might also have a big impact on retention.*

[Clinician 1]

Second, participants stressed the importance of the information being conveyed in an understandable manner. AI experts often spoke about the “language barrier” they experience when communicating about AI with clinicians, and clinicians confirmed this gap when they remarked on developer jargon. Participants suggested communicating in statistical language, such as *P* values or false positives, which is generally more in line with clinicians’ education and academic training. Others suggested shrinking this “language barrier” by providing general AI training for clinicians, for example, from the hospital or during medical training. Another familiar way of providing information would be in the form of papers, which clinicians are accustomed to reading. The TRIPOD-AI example was well received by participants, as its resemblance to the layout of traditional academic papers appeared to create a sense of familiarity. However, some clinicians stressed that they then might as well read the entire paper, while AI experts showed significant information was still missing due to word count restraints.

*I think that, in any case, education about AI will simply be necessary for everyone in the near future, because it’s gradually being used more and more in medicine. And it’s important to understand how it works or how something like that even comes about.*

[Clinician 3]

Third, clinicians often labeled themselves as being intrigued by AI and intrinsically motivated to gather information about new technologies. However, many clinicians also stressed that they themselves or their coworkers would not have enough time to read up about a model. This shows a difference in needs between clinicians, which calls for customizable or layered information provision. An example of how this works out in practice would be through providing information blocks or pop-ups on demand or linking to (validation) papers and other useful resources.

*And references – well, of course people aren’t going to look at them very much either, so they quickly become less important. And people don’t have endless time.*

[Clinician 6]

Finally, of all the examples, the Model Card example was perceived as the least desired method by the participants, while the first page of the Model Facts was the most appreciated. However, according to participants, Model Facts still missed essential information blocks that were provided in the TRIPOD-AI, such as class imbalance, inclusion and exclusion criteria, and population size. Participants recommended that this information be integrated as much as possible within the software to use the AI-CDSS.

*Well, if you want to know some of the background, then the TRIPOD would be my preference. But if it’s purely about how to apply it in the clinic, then the Model Facts is fine – it’s a bit shorter and more focused on clinical applicability.*

[Clinician 2]

## Discussion

This qualitative study explored the informational needs and preferences of clinicians for understanding and appropriately using AI-CDSS through semistructured interviews. Besides, this study explored AI experts’ perspectives on what information should be communicated to clinicians to enable safe and appropriate use of AI-CDSS. From the thematic analysis, four key domains were identified that the participants considered essential: (1) understanding the population the model was trained on, (2) interpreting performance metrics in a clinically meaningful way, (3) being clearly informed about limitations and boundaries of use, and (4) having the information presented in an accessible and integrated format. Elements of the presented Model Facts, Model Card, and TRIPOD-AI examples were seen as valuable in addressing these needs, though none were considered fully sufficient on their own.

The first aspect concerns the ability to assess whether the patient in front of the clinician matches the training population of the AI-CDSS. Information on the data’s origin, distribution, and size was thus seen as crucial to avoid inappropriate extrapolation and potential harm. This aligns with guidelines made for AI in clinical research in which proper use of predictive models increases when end users understand the AI models better [28,29]. At the time of this writing, many technical solutions aim to address dataset limitations, such as undersampling in the presence of class imbalances [38]. However, little research exists on how to equip end users to recognize and act upon known data limitations in real-time use. The TRIPOD-AI example was praised by participants for including elements like class imbalance and inclusion and exclusion criteria. However, participants suggested more use of visual tools or interactive comparison modules, which reflects a desire for more transparency delivered in comprehensive formats.

Additionally, there was a need to understand model performance in ways that directly inform clinical decision-making. Although clinicians appreciated seeing performance metrics, like the one shown in the Model Facts table, they found that metrics such as the AUC lacked context. This was also the case in the study performed by Frey et al [39], in which the Model Facts and the table containing information on performance and validation were not understood. These concerns are in line with other existing literature that argues for optimizing performance reporting to the clinical context and translating technical metrics into practical terms [40]. Instead, participants preferred metrics linked to treatment-relevant thresholds. Several AI experts also pointed out that communicating uncertainty is vital for identifying edge cases where model performance might be unreliable. Performance should therefore not only be reported on a global level but also in ways that match the decision points clinicians face.

Another key finding concerned the need for transparent warnings and clear boundaries for appropriate model use. While participants acknowledged that standards such as TRIPOD-AI and Model Facts do include these elements, these sections are often too vaguely phrased, making them less actionable for clinicians. The importance of clear, structured warnings is well established in other areas of health care, for example, in drug labels, which provide standardized information on indications, contraindications, and potential risks [41]. Such an approach would move beyond abstract disclaimers, offering clinicians tangible guidance to support responsible decision-making. Besides, some participants proposed integrating restrictions directly into the software, like locking out-of-scope inputs or showing warnings when the model is applied beyond its intended population. However, these suggestions were met with mixed reactions due to concerns about alert fatigue, which is an already frequently experienced mental strain in clinical decision support tools [42,43].

Finally, participants focused on how the information is structured and delivered to the end user. Participants advocated for layered information design, in which summary-level information is accessible at first glance, with additional detail available on demand [44]. This design principle is widely used in both health care settings, for example, in patient decision aids [45,46], and in other industries or webpages, like Wikipedia (Wikimedia Foundation). Interactive data visualizations were also suggested for understanding how individual patients compare to the broader training dataset. This preference mirrors trends in other industries, such as finance and data analytics, where interactive visual tools enhance sense-making and decision quality [47]. Clinicians also emphasized the importance of integrating information in the AI-CDSS interface and supporting information dissemination through workshops or e-learning modules. While the Model Facts format was seen as more accessible and clinically relevant, it lacked essential components, including training data information, which were better covered in TRIPOD-AI.

In general, participants throughout this study consistently emphasized that AI-CDSS can only be made safe, effective, and clinically relevant through co-creation with clinicians at every stage of development and deployment. This would start with jointly defining the model’s purpose, identifying which clinical decisions it should support, and determining what constitutes a “good enough” model in clinical practice. Such early alignment likely helps to determine whether the model’s goals are both clinically valuable and technically feasible. Beyond initial development, it seems that clinicians should also be involved in designing the model to fit existing workflows, identifying potential limitations, and establishing appropriate safeguards. AI experts noted they cannot define these elements alone because these require clinical insight. While the value of co-creation in AI development is well recognized in theory, it remains inconsistently practiced [48,49]. One approach formalizing co-creation is design thinking, which is a user-centered, iterative process that emphasizes empathy, problem definition, rapid prototyping, and testing in real-world contexts [50]. Embedding such methods more systematically into AI development is essential to close the gap between technical promise and clinical utility [51].

Although this study was rooted in the Dutch tertiary care context, the findings likely extend to other modernized health care systems preparing for AI implementation. Particularly, these findings are transferable within Europe, given the European AI Act’s mandate for ensuring AI literacy among professional users. This regulation requires end users across all member states to be competent in using high-risk AI systems [52]. The educational and informational needs identified here are directly relevant to compliance efforts across the continent. Beyond the regulatory context, the core themes reflect universal medical concerns, extending the study’s relevance to other modernized health care systems globally.

The results of this study should be interpreted in light of several limitations. First, the use of convenience sampling may have introduced selection bias, as individuals who voluntarily participate in research are often more engaged with and positive about the topic. Second, while the study aimed to explore clinicians’ informational needs regarding AI-CDSS, many clinicians seemed to lack in-depth understanding of AI, which may have limited their ability to fully articulate their needs. To address this, perspectives from AI experts were included to enrich the findings. Third, the interdisciplinary approach introduced heterogeneity, due to participants coming from a range of professional backgrounds or clinical specialties. While this breadth is a strength, allowing for a more comprehensive understanding of cross-disciplinary needs, it may also obscure discipline-specific differences. For example, certain formats or tools may be more relevant or familiar to specific specialties. Fourth, the examples of AI model documentation were presented to all participants in a fixed order. The lack of randomization in the sequence may have introduced order effects, such as primacy bias, which could have influenced comparative feedback. Future research should explore how disciplinary context shapes informational needs and preferences, ideally through larger-scale studies using quantitative methods that allow for broader demographic and professional representation and more robust comparisons across subgroups.

In conclusion, when using AI in clinical practice, it is important that clinicians use these technologies in a safe and informed way. However, this information provision likely falls short in meeting their informational needs, contributing to the lack of large-scale adoption. AI-CDSS developers should clearly communicate both who the model is for, including characteristics of its training data, and how it performs in clinically meaningful terms. This must include explicit limitations and appropriate warnings, presented in a way that is understandable, visual, and ideally interactive. While existing formats such as Model Cards, Model Facts, and TRIPOD-AI offer valuable starting points, none fully meet these needs on their own. Future efforts should consider combining and adapting elements from these formats to better support clinical use. Effective integration of AI-CDSS requires collaboration with clinicians during development and targeted education during implementation. Addressing informational needs of clinicians is critical to ensure appropriate use and supports the safe and effective integration of AI-CDSS into clinical practice.

## Supplementary material

10.2196/85228 Multimedia Appendix 1 Interview guide clinicians.

10.2196/85228 Multimedia Appendix 2 Interview guide artificial intelligence (AI) experts.

10.2196/85228 Multimedia Appendix 3 Examples of Model Card, Transparent Reporting of a multivariable prediction model for Individual Prognosis Or Diagnosis–Artificial Intelligence (TRIPOD-AI), and Model Facts.

10.2196/85228 Multimedia Appendix 4 Clusters with themes clinicians.

10.2196/85228 Multimedia Appendix 5 Clusters with themes artificial intelligence (AI) experts.

10.2196/85228 Checklist 1 Consolidated Criteria for Reporting Qualitative Research (COREQ) checklist.

## Abbreviations

AI: artificial intelligence
AI-CDSS: artificial intelligence-driven clinical decision support systems
AUC: area under the curve
CDS: Clinical Data Science
CONSORT-AI: Consolidated Standards Of Reporting Trials–Artificial Intelligence
COREQ: Consolidated Criteria for Reporting Qualitative Research
DECIDE-AI: Developmental and Exploratory Clinical Investigation of Decision support systems driven by Artificial Intelligence
FHML-REC: Faculty of Health, Medicine, and Life Sciences–Research Ethics Committee
SPIRIT-AI: Standard Protocol Items: Recommendations for Interventional Trials–Artificial Intelligence
TRIPOD-AI: Transparent Reporting of a multivariable prediction model for Individual Prognosis Or Diagnosis – Artificial Intelligence
