# Supplementary material for: Understanding Clinicians’ Informational Needs for AI-Driven Clinical Decision Support Systems: Qualitative Interview Study
Source: JMIR Med Educ. 2026 Mar 12;12:e85228. doi: 10.2196/85228 (PMC12989292; doi:10.2196/85228)
Supplement: Multimedia Appendix 1 [file mededu-v12-e85228-s001.docx]

Appendix 1 – Interview guide clinicians

| Opening |  | Goodmorning / afternoon / evening. My name is Simone Mingels from Maastricht University. I conduct interviews with clinicians and AI experts in order to learn about the informational needs of clinicians concerning AI in clinical decision support systems, such as decision-aids and prediction models. |
| --- | --- | --- |
|  |  | During the interview I will ask you about your experience with AI within your job, and in which way you gain information about these AI models. After I will ask you which informational needs you have, which we will also discuss using a few examples. |
|  |  | Your insights will contribute towards a better understanding of informational needs for AI in clinical practice. |
|  |  | All information we gather within this interview will be confidential. We appreciate your help, the interview will take between 30 and 45 minutes to complete. The interview consists out of 4 parts, with a total of about 15 questions. |
|  |  | |
| Consent for recording |  | You received and signed the informed consent we provided you with. By signing this you gave permission for recording this interview. Is this permission still granted? If at any moment you would want to stop participating in this research you can inform me. Are there any questions before starting the recording? Questions can be asked anytime during the interview as well. |
|  |  | |
| Part 1: Participant characteristics (2 min) |  | |
|  | 1.1 | What is your age? |
|  | 1.2 | What is your specialism? |
|  | 1.3 | How long have you been working (within this specialism)? |
|  |  | |
| Part 2: Experience with AI (10 min) |  | |
|  | 2.1 | Can you tell me what you already know about AI for the medical field? |
|  | 2.2 | Do you have experience with AI yourself? |
|  | 2.3 | Have you ever used an AI model for making a diagnosis- or treatment decision with a patient? |
|  |  | *Before the interviews we should check the existing decision aids and prediction models in the specialism of the clinician used in clinical practice in order to give examples.* |
|  | 2.4 | Which information did you receive from the developers of the system before using the model? |
|  | 2.5 | Did you miss information in the beginning which you needed (after using it)? |
|  |  | |
| Part 3: informational needs (10 min) |  | |
|  | 3.1 | Which information would you have wanted when you would receive a new AI model? |
|  | 3.2 | How would you like to receive this information? |
|  |  | *For example in the system itself, on a (physical) card, per mail, an information session, etc..* |
|  | 3.3 | When would you like to receive this information? |
|  |  | *For example before you would use it, available at all times, automatic alarms in the system, etc...* |
|  |  | |
| Part 4: Examples (20 min) | 4 | Showing the filled in versions of model facts, model cards, tripod-AI |
|  |  | *In case of a face-to-face interview, physical copies will be taken to the interview and clinicians can scribble on these copies and point out what information would and wouldn't be useful. The researcher or clinician should point out aloud about which parts they are talking, so it can be written down in the transcript.* |
|  |  | *in case of an online interview, clinicians will be shown the information by sharing our screen and discussing it.* |
|  | 4.1 | What is your opinion on these examples? Which standard has your preference? Why? |
|  | 4.2 | Would you appreciate receiving this information with an AI model? |
|  | 4.3 | Why would or wouldn't you appreciate receiving this information? |
|  | 4.4 | Is this information described in an understandable way? Is the language used understandable? |
|  | 4.5 | To which depth would you want to receive information? (based on the examples) |
|  | 4.6 | What information do you think your coworkers would need? Do you think they need an equal amount of information? |
|  |  | |
| Conclusion |  | This was the last question of this interview. Are there anymore remarks or questions regarding this interview or the research? |
|  |  | I would like to thank you for your participation in this interview. After completing all interviews I plan to analyze the transcripts and use the outcomes of the interviews to construct a questionnaire. The goal is to distribute this questionnaire to a broader setting in order to see whether the informational needs described in the interviews are shared by other clinicians as well. |
|  |  | I would like to verify my findings and the constructed questionnaire made based on these interviews with you to see whether i have interpreted this interview correctly. Would you be open to be reached out to again in a couple of months? |
|  |  | I would like to thank you for participating in this research and i wish you a good day. |
