# Supplementary material for: Understanding Clinicians’ Informational Needs for AI-Driven Clinical Decision Support Systems: Qualitative Interview Study
Source: JMIR Med Educ. 2026 Mar 12;12:e85228. doi: 10.2196/85228 (PMC12989292; doi:10.2196/85228)
Supplement: Multimedia Appendix 2 [file mededu-v12-e85228-s002.docx]

Appendix 2 – Interview guide AI experts

| Opening |  | Goodmorning / afternoon / evening. My name is Simone Mingels from Maastricht University. I conduct interviews with clinicians and AI experts in order to learn about the informational needs of clinicians concerning AI in clinical decision support systems, such as decision-aids and prediction models. |
| --- | --- | --- |
|  |  | During the interview I will ask you about your experience with AI within your job, and in which way you gain information about these AI models. After I will ask you which informational needs you have, which we will also discuss using a few examples. |
|  |  | Your insights will contribute towards a better understanding of informational needs for AI in clinical practice. |
|  |  | All information we gather within this interview will be confidential. We appreciate your help, the interview will take between 30 and 45 minutes to complete. The interview consists out of 4 parts, with a total of about 15 questions. |
|  |  | |
| Consent for recording |  | You received and signed the informed consent we provided you with. By signing this you gave permission for recording this interview. Is this permission still grasnted? If at any moment you would want to stop participating in this research you can inform me. Are there any questions before starting the recording? Questions can be asked anytime during the interview as well. |
|  |  | |
| Part 1: Participant characteristics |  | |
|  | 1.1 | What is your age? |
|  | 1.2 | What is your specialism? |
|  | 1.3 | How long have you been working (within this specialism)? |
|  |  | |
| Part 2: Experience with AI |  | |
|  | 2.1 | Have you ever made an AI-model for medical practice? |
|  | 2.2 | If yes, which goal and function did this AI-model have? |
|  | 2.3 | What kind of AI-model was this? |
|  |  | *For example: a deep learning model, random forest model, logistic regression etc.* |
|  | 2.4 | Why did you choose to construct this kind of AI-model? |
|  | 2.5 | How did you report the performance and background information of this model? |
|  |  | *for example, did you use a standard for reporting in research, describe it in a paper, use model cards, etc..* |
|  | 2.6 | Do you have experience with reporting standards? (Such as TRIPOD, CONSORT, Model cards) |
|  | 2.7 | Did you have to explain the AI-model you constructed to endusers (clinicians)? |
|  |  | |
| Part 3: informational needs |  | |
|  | 3.1 | Which information do you think is crucial to receive for clinicians before using an AI-model? |
|  | 3.2 | What information could you provide clinicians? |
|  |  | *For example, think about making a guide, model cards / facts, etc.* |
|  | 3.3 | When are you willing and able to provide this information? |
|  |  | *For example before you would use it, available at all times, automatic alarms in the system, etc...* |
|  |  | |
| Part 4: Examples | 4 | Showing the filled in versions of model facts, model cards, tripod-AI |
|  |  | *In case of a face-to-face interview, physical copies will be taken to the interview and clinicians can scribble on these copies and point out what information would and wouldn't be useful. The researcher or clinician should point out aloud about which parts they are talking, so it can be written down in the transcript.* |
|  |  | *in case of an online interview, clinicians will be shown the information by sharing our screen and discussing it.* |
|  | 4.1 | What is your opinion on these examples? Which standard has your preference? Why? |
|  | 4.2 | Which information do you think clinicians need to correctly use AI-models? |
|  | 4.3 | Is all information you think is of importance reported within this standard? |
|  | 4.4 | Which information needs to minimally be included in the report? |
|  | 4.5 | What do you think that your coworkers think of these reporting standards? |
|  |  | |
| Conclusion |  | This was the alst question fo this interview. Are there anymore remarks or questions regarding this interview or the research? |
|  |  | I would like to thank you for your participation in this interview. After completing all interviews I plan to analyse the transcripts and use the outcomes of the interviews to construct a questionnaire. The goal is to distribute this questionnaire to a broader setting in order to see whether the informational needs described in the interviews are shared by other clinicians as well. |
|  |  | I would like to thank you for participating in this research and i wish you a good day. |
