# Supplementary material for: Understanding Clinicians’ Informational Needs for AI-Driven Clinical Decision Support Systems: Qualitative Interview Study
Source: JMIR Med Educ. 2026 Mar 12;12:e85228. doi: 10.2196/85228 (PMC12989292; doi:10.2196/85228)
Supplement: Multimedia Appendix 3 [file mededu-v12-e85228-s003.docx]

Appendix 3 – Examples of Model Card, Transparent Reporting of a multivariable prediction model for Individual Prognosis Or Diagnosis–Artificial Intelligence (TRIPOD-AI), and Model Facts

Developed from:

- Fang H, Miao H. Introducing the Model Card Toolkit for Easier Model Transparency Reporting. GoogleResearch. Published online July 2020.
- Mitchell M, Wu S, Zaldivar A, et al. Model cards for model reporting. In: Association for Computing Machinery, Inc; 2019:220-229. doi:10.1145/3287560.3287596

Model Card for Estimating Treatment Effect in Elderly Patients With Stage III Colon Cancer

| **Summary**  While adjuvant therapy with capecitabine and oxaliplatin (CAPOX) has been proven to be effective in stage III colon cancer, capecitabine monotherapy (CapMono) might be equally effective in elderly patients. Unfortunately, the elderly are under-represented in clinical trials and patients included may not be representative of the routine care population. Observational data might alleviate this problem but is sensitive to biases such as confounding by indication. Here, we build causal models using Bayesian Networks (BNs), identify confounders, and estimate the effect of adjuvant chemotherapy using survival analyses. | |
| --- | --- |
| **Model details** | |
| Developed by | First – Last name |
| Funded by | AstraZeneca (Inst), Roche (Inst), Netherlands Comprehensive Cancer Organisation (Inst), QP&S/RanD (Inst), Varian Medical Systems (Inst), Philips Healthcare (Inst), OncoRadiomics (Inst) |
| Model type | Bayesian Networks (BNs) with Necessary Path Condition (NPC), Silander-Myllymaki (SM), Max-Min Hill-climbing (MMHC) algorithms |
| Language(s) | R & Python |
| License | Not applicable |
| **Model sources** | |
| Repository | *github link* |
| Paper | *link to paper* |
| Demo | Not applicable |
| **Uses** | |
| Direct use | The model can be used on elderly patients (70 years or older) diagnosed with stage III colorectal cancer to estimate the treatment effects. Patient treatment should involve surgery, but they should not have received adjuvant chemotherapy or only CAPOX or CapMono. |
| Downstream use | This model is trained on data from patients located in the southeastern part of the Netherlands. If this model is used in different areas of the Netherlands, it should be tested. When this model would be used in different countries, national treatment guidelines should be reviewed compared to this model |
| Out-of-Scope Use | The model should not be used in a population with a different (colon cancer stage) diagnosis or patients that are younger than 70 years old. Besides, this model cannot be used for patients that have already received other adjuvant cancer treatments besides CAPOX or CapMono |
| **Bias, Risks, and Limitations** | |
| Limitations | This model was not externally validated |
| Recommendations | A new user of this model should validate this model before implementation |
| **Training details** | |
| Training data | Exported from the Netherlands Cancer Registry (NCR) |
| Inclusion of patients: | - Diagnosed between 2005 and 2012 - Pathologic stage III colon cancer - 70 years or older - Southeastern part of the Netherlands |
| Exclusion of patients | - Who died within 90 days of surgery (N=125) - Received chemotherapy other than CAPOX or CapMono |
| Final dataset | 982 records |
| Variables | Available information of NCR:   - Sex - Age - ASA classification - pT - pN - Tumor subsite (coded according to ICD-0-3) - Differentiation grade   Additional variables acquired from medical records   - Adjuvant therapy - Number of comorbidities - Development of (local) recurrence |
| **Training procedure** | |
| Preprocessing | Age was discretized into 3 categories: 70-74, 75-79, and 80 years and older. The anatomic subsite was relabeled as proximal, distal or unknown/unspecified. Overall survival counted from the date of surgery, was discretized into 5 Boolean variables representing 1- to 5-year survival |
| Training hyperparameters | Search algorithm – Hill Climbing  Scoring function – BIC  Maximal parents – 3  Initial structure - none |
| Speeds, sizes, times | Unknown |
| **Evaluation** | |
| Testing data | 10% of the included dataset |
| Factors | New treatments such as surgeries or medications can have impact on the survival or recurrence for patients within this group. This is why the model might predict less accurate in the future |
| Metrics | See training hyperparameters |
| Results | AUC = 0.88  PPV @ sensitivity of 60% = 0.14 |
| Summary | TBD |
| **Environmental impact** | |
| Technical specifications | Unknown |
| Model architecture & objective | Unknown |
| Compute infrastructure | Unknown |
| Hardware | Unknown |
| Software | Unknown |
| **Additional information** | |
| Citation | *Link to paper* |
| Glossary | *Link to paper* |
| More information | *Link to paper* |
| Model Card Authors & contact | *email* |

Developed from :

| 1. Collins GS, Reitsma JB, Altman DG, Moons KG. Transparent reporting of a multivariable prediction model for individual prognosis or diagnosis (TRIPOD): the TRIPOD Statement. BMC Medicine. 2015;13(1):1. doi:10.1186/s12916-014-0241-z |
| --- |
| 1. Collins GS, Dhiman P, Andaur Navarro CL, et al. Protocol for development of a reporting guideline (TRIPOD-AI) and risk of bias tool (PROBAST-AI) for diagnostic and prognostic prediction model studies based on artificial intelligence. BMJ Open. 2021;11(7). doi:10.1136/bmjopen-2020-048008 |
| 1. Collins GS, Moons KGM, Dhiman P, et al. TRIPOD+AI statement: updated guidance for reporting clinical prediction models that use regression or machine learning methods. BMJ. Published online April 16, 2024:e078378. doi:10.1136/bmj-2023-078378 |

TRIPOD-AI for Estimating Treatment Effect in Elderly Patients with Stage III Colon Cancer

| **Item** | **Section/Topic** | **Information** |
| --- | --- | --- |
| 1 | Title | Estimating Treatment Effect in Elderly Patients with Stage III Colon Cancer |
| **Introduction** | | |
| 2 | Background | Adjuvant chemotherapy in elderly is effective although previous analyses dispute the benefit of adding oxaliplatin to fluoropyrimidines. Using observational data to estimate treatment effect always comes with the risk of bias, specifically confounding by indication. |
| 3 | Objectives | We use structure learning algorithms for Bayesian Networks, in conjunction with clinical knowledge, to identify confounders, mitigate this risk, and reliably estimate the effect of adjuvant treatment in colon cancer. |
| **Methods** | | |
| 4 | Data | The source of data is in the population-based Netherlands Cancer Registry (NCR), specifically from the Eindhoven area. Information on patient and tumor characteristics were extracted from the medicsl records. The quality of the data is high, due to thorough training of the registration team and computerized consistency checks at regional and national levels. In 2013 and 2013, details regarding adjuvant therapy, the number of comorbidities, and development of (local) recurrence was acquired from medical records and added to the NCR. Vital status was updated until January 2021. |
| 5 | Participants | Inclusion criteria:   - 70 years and older - Diagnosed with pathologic stage III (pT_1-4_N_1-2_M_0_) colon cancer     Exclusion criteria:   - Died within 90 days after surgery - Receiving chemotherapy other than CAPOX or CapMono     Treatment with:   - Surgical resection - Surgical resection followed by adjuvant capecetabine + oxaliplatin - Surgical resection followed by adjuvant capecetabine only |
| 6 | Data preparation | Age was discretized into three categories: 70-74, 75-79, 80 years and older. The anatomic subsite was relabeled as proximal, distal or unknown/unspecified. Overall survival countred from the data of surgery, was discretized into 5 Boolean variables representing 1- to 5-year survival |
| 7 | Outcome | - Overall survival for 1, 2, 3, 4 and 5 years - Recurrence at 1 and 2 years |
| 8 | Predictors | - Sex - Age - ASA classification - Pathologic stage (pT) - Pathological lymph node classification (pN) - Tumor subsite (coded according to ICD-0-3) - Differentiation grade |
| 9 | Sample Size | 982 records |
| 10 | Missing data | Not applicable for patients who had a follow-up of <5 years and were alive at the time of follow-up |
| 11 | Analytical methods | Bayesian network |
| 12 | Class Imbalance | Sex: Male (45.1%); Female (54.9%)  Age: 70-74 (17.1%); 75-79 (31.3%); ≥80 (51.6%)  Comorbidities: none (15.4%); 1 (21.4%); ≥2 (59.8%); unknown (3.3%)  ASA: 1 (2.5%); 2 (38.3%); 3 (34.9%); 4 (0.6%); unknown (23.7%)  pT: T1 (1.6%); T2 (8.1%); T3 (71.7%); T4 (18.6%)  pN: N1 (75.9%); N2 (24.1%)  Location: Proximal (62.4%); Distal (36.5%); Other (1.1%)  Grade: g1 (5.4%); g2 (62.5%); g3 (25.7%); g4 (0.2%); unkown (6.2%) |
| 13 | Fairness | Unknown |
| 14 | Model output | - Overall survival for 1, 2, 3, 4 and 5 years - Recurrence at 1 and 2 years |
| 15 | Training vs Evaluation | Unknown |
| 16 | Ethical approval | In the Netherlands, studies with anonymized patient records do not fall under the scope of the Medical Research Involving Human Subjects Act. This study is therefore exempt from medical ethics review. |
| 17 | Open Science | |
| 17.a | Funding | AstraZeneca (Inst), Roche (Inst), Janssen (Inst), Netherlands Comprehensive Cancer Organisation (Inst), QP&S/RanD (Inst), Varian Medical Systems (Inst), Philips Healthcare (Inst), OncoRadiomics (Inst) |
| 17.b | Conflict of Interest | No other potential conflicts of interest were reported |
| 17.c | Protocol | Unknown |
| 17.d | Registration | Unknown |
| 17.e | Data sharing | *link to paper* |
| 17.f | Code sharing | *github link* |
| 18 | Patient & Public involvement | Unknown |
| **Results** | | |
| 19 | Participants |  |
| 20 | Model development | Algorithms for Bayesian Network: Necessary Path Condition (NPC), Max-Min Hill-Climbing (MMHC), Silander-Myllymaki (SM).  The level of significance was 0.05 |
| 21 | Model specification | Bayesian Network with 2 nodes for recurrence at 1 and 2 years  Bayesian Network with 5 nodes for overall survival |
| 22 | Model performance | AUC = 0.88%  PPV @ sensitivity of 60% = 0.14% |
| 23 | Model updating | Unknown |
| **Discussion** | | |
| 24 | Interpretation | The strong effect of adjuvant therapy on survival in the cohort was found |
| 25 | Limitations | No external validation |
| 26 | Usability of the model in the context of current care | The models can be further used for the survival prediction in the described patient cohort |

Developed from:

Sendak MP, Gao M, Brajer N, Balu S. Presenting machine learning model information to clinical end users with model facts labels. npj Digital Medicine. 2020;3(1). doi:10.1038/s41746-020-0253-3

Model facts for Estimating Treatment Effect in Elderly Patients with Stage III colon cancer

| *Model facts* | **Model name:** Estimating Treatment Effect in Elderly Patients with Stage III Colon Cancer | | **Locale:** Netherlands Comprehensive Cancer Organisation |
| --- | --- | --- | --- |
| **Approval Date:** 04/03/2024 | | **Last Update:** 05/12/2023 | **Version:** 1.4 |
| **Summary:**  While adjuvant therapy with capecitabine and oxaliplatin (CAPOX) has been proven to be effective in stage III colon cancer, capecitabine monotherapy (CapMono) might be equally effective in elderly patients. Unfortunately, the elderly are under-represented in clinical trials and patients included may not be representative of the routine care population. Observational data might alleviate this problem but is sensitive to biases such as confounding by indication. Here, we build causal models using Bayesian Networks (BNs), identify confounders, and estimate the effect of adjuvant chemotherapy using survival analyses. | | | |
| **Mechanisms** | | | |
| Outcome | | Estimation of the treatment effect of an elderly patient with Stage III colon cancer | |
| Output | | - Overall survival for 1, 2, 3, 4 and 5 years - Recurrence at 1 and 2 years | |
| Target population | | Elderly patients (70 years or older) with Stage III Colon Cancer with surgery in their treatment plan, but have not yet received adjuvant chemotherapy or only CAPOX or CapMono | |
| Time of prediction | | Before deciding for adjuvant chemotherapy | |
| Input Data source | | Electronic Health Record (EHR) | |
| Input Data type | | Sex, Age, ASA classification, pT, pN, Differentiation grade, Adjuvant therapy, number of comorbidities | |
| Training data location & time-period | | Cohort of patients diagnosed in the southeastern part of the Netherlands between 2005 and 2012 with pathologic stage III colon cancer and 70 years or older. Selected from the Netherlands Cancer Registry (NCR). Additional information was gathered in 2013-2014 and added to the NCR. Vital status was updated until January 2021. | |
| Model type | | Bayesian Networks (BNs) with Necessary Path Condition (NPC), Silander-Myllymaki (SM), Max-Min Hill-climbing (MMHC) algorithms | |
| **Validation & Performance**   \|  \| **AUC** \| **PPV @ sensitivity of 60%** \| **Sensitivity @ PPV of 20%** \| **Population tested** \| **Cohort URL/DOI** \| \| --- \| --- \| --- \| --- \| --- \| --- \| \| **Local retrospective** \| 0.88 \| 0.14 \| 0.50 \| The Netherlands \| **link** \| \| **Local Temporal** \| 0.90 \| 0.20 \| 0.60 \| The Netherlands \| **link** \| \| **Local Prospective** \| **TBD** \| **TBD** \| **TBD** \| **TBD** \| **TBD** \| \| **External** \| **TBD** \| **TBD** \| **TBD** \| **TBD** \| **TBD** \| \| **Target population** \| 0.94 \| 0.20 \| 0.60 \| The Netherlands \| **link** \|   **.** | | | |
| **Uses and Directions** | | | |
| Benefits | | Estimating treatment effects can assist making more informed treatment decisions | |
| Target population & use case | | Elderly patients (70 years or older) with diagnosed pathologic stage III colon cancer | |
| General use | | This model is intended to be used by clinicians to estimate treatment effects on patients to assist the treatment decision making. The model is not a diagnostic tool for colon cancer and is not meant to guide or drive clinical care. This model is intended to complement other pieces of patient information related to treatment decision making | |
| Appropriate decision support | | The model estimates the treatment effects of an elderly patient with diagnosed pathologic stage III colon cancer. The clinician (together with the patient) can make a treatment decision based on the expected recurrence or survival. | |
| Before using this model | | Test the model retrospectively and prospectively on a cohort that reflects the target population that the model will be used upon to confirm validity of the model within a local setting | |
| Safety & efficacy evaluation | | Analysis of data from clinical trial is underway | |
| **Warnings** | | | |
| Risks | | Even if used appropriately, the prediction from this model is an estimation, and it gives a chance for recurrence and survival. Patient preferences and characteristics should be considered when making a treatment decision. | |
| Inappropriate Settings | | This model was not trained or evaluated on patients with a different diagnosis or in a different age category. Do not use this model in a different setting without further evaluation | |
| Clinical Rationale | | The model is not interpretable and does not provide rationale for its estimation. Clinical end users are expected to place model output in context with other clinical information to make final treatment decisions | |
| Inappropriate decision support | | This model may not be accurate outside of the target population which are elderly patients with stage III colon cancer in the Netherlands | |
| Generalizability | | This model was primarily evaluated on the older population within the Netherlands, do not use this model for younger patients or in other countries without further evaluation | |
| Discontinue use if | | Clinical staff raise concerns about utility of the model for the indicated use case or large, systematic changes occur at the data level that necessitates re-training of the model | |
| **Other information** | | | |
| Outcome Definition | | *link to paper* | |
| Related model | | *link to paper * | |
| Model development & validation | | *link to paper * | |
| Model implementation | | *link to paper * | |
| Clinical Trial | | *link to paper * | |
| Clinical impact evaluation | | *link to paper * | |
| For inquiries and additional information | | Please email *name developer* - *email* | |
