# Supplementary figures and images for: Understanding Clinicians’ Informational Needs for AI-Driven Clinical Decision Support Systems: Qualitative Interview Study
Source: JMIR Med Educ. 2026 Mar 12;12:e85228. doi: 10.2196/85228 (PMC12989292; doi:10.2196/85228)

# Multimedia Appendix File 4 – Clusters with themes Clinicians


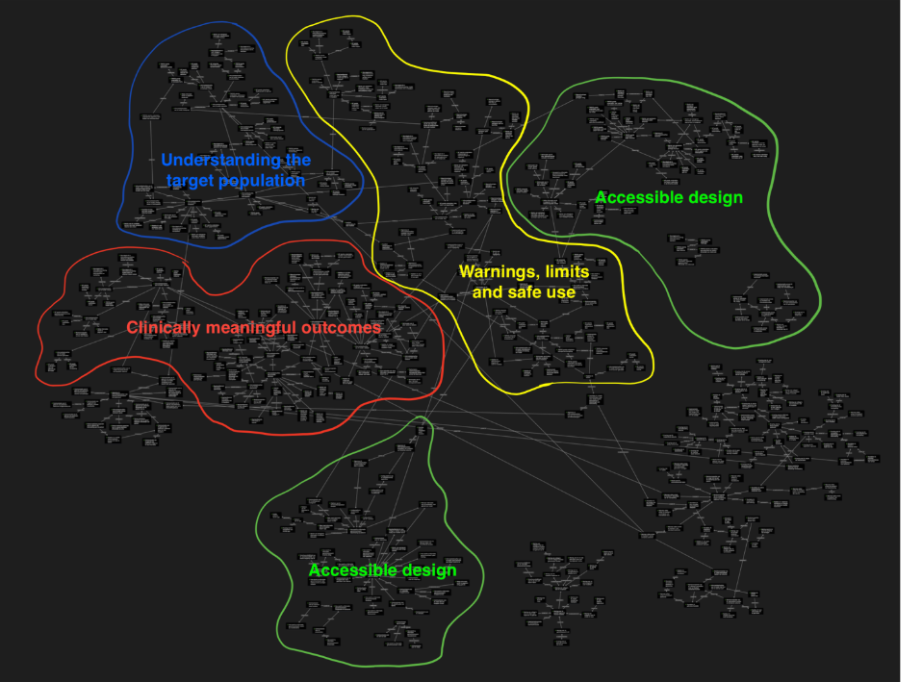

Supplement: Multimedia Appendix 4 [file mededu-v12-e85228-s004.docx]

# Multimedia Appendix 5 – Clusters with themes AI experts


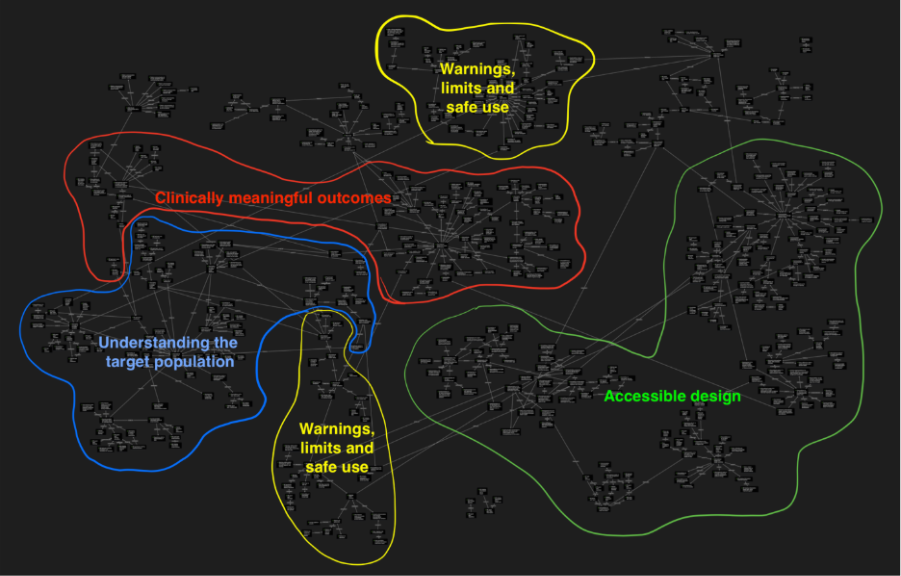

Supplement: Multimedia Appendix 5 [file mededu-v12-e85228-s005.docx]
